# Progress of Diseases of the Throat and Nose

**Published:** 1902-01-18

**Authors:** 


### Progress of Diseases of the Throat and Nose.

(*Continued from page* 254.)

**Œdema Glottidis.**—Somers,[11] in an article on the use of suprarenal gland in peritonsillar abscess, refers to the great usefulness of the same agent in cases of œdema glottidis with dyspnœa. He states that several cases appear in which striking results were obtained in œdema of the glottis, and especially notable was the case seen by H. H. Curtis,[12] in which he was of the opinion that without the use of the adrenal, tracheotomy would have been necessary. "Such a case has recently come under my observation in private practice in which there was not the least shadow of doubt that the adrenal saved the patient from a serious operation and dissipated the glottic œdema which was rapidly inducing suffocation." Similarly, as regards the presence of œdema, was Boeker's case,[13] in which acute œdema developed suddenly during erysipelas. Six grains of the desiccated gland were placed on the patient's tongue and allowed to dissolve, with the result that decided amelioration occurred in five minutes and uninterrupted recovery followed.

**Tuberculosis of the Larynx.**—An interesting and valuable résumé of the present position on this question has been made by Sendziak.[14] The direct treatment of tuberculosis of the larynx commenced with the introduction of the laryngoscope in the year 1858, and was commenced by Turck and Czermatt. In 1880 Moritz Schmidt introduced the modern methods, while in 1885, a year after the discovery of cocaine, Professor Krause used with success the treatment by lactic acid applications to the diseased areas. Then M. Schmidt and Heryng developed the method of first curetting the tuberculous deposits, followed by lactic acid applications. In the year 1890 Professor Schroetter stated that in his opinion we are never able to remove so completely all that is pathologically affected as to prevent recurrences, and that it is not every larynx that can tolerate such energetic treatment. But from the time that Heryng described a case in 1887, the curability of laryngeal phthisis was definitely solved; moreover, clinical observation has more than once proved the possibility of complete recovery from this disease, both by *spontaneo modo* as well as, still more readily, under the influence of suitable treatment. On the other hand, it must be admitted that recoveries are exceedingly rare, and that certain favourable conditions are necessary, and of these a predisposition of the organism to the production of fibrous tissue in the lungs, as well as in the larynx, is most essential.

Sendziak goes on to state that the local therapeutic measures may be grouped in three divisions—the milder remedies, the more active, and the palliative. The milder remedies we use in the initial stages, *e.g.*, localised catarrhal inflammation—remedies also useful in the later stages of ulcerations. Amongst these remedies orthoform has a high place in the author's estimation. And as regards the more active measures, Sendziak is in agreement with most laryngologists in giving lactic acid application the first place; but it is essential that we choose the cases suitable for the treatment and that it should be applied rationally. Begin with strong solutions—at least 50 per cent.—and quickly pass to the pure lactic acid. Generally the acid must be applied energetically by rubbing, and until a brown coloration of the surface is produced by the lactic acid coming in contact with the blood. It is also of importance that the intervals between the applications of the acid should be of sufficient length (*i.e.*, one or two weeks), to allow the complete separation of the dead epithelium. While agreeing that the most suitable cases are those in which ulceration has taken place, Sendziak has observed favourable results also in cases of tuberculous infiltration, especially of the epiglottis. He is not in favour of the submucous injection of the acid, as recommended by Heryng, Hagen, and Gleitsmann. In suitable cases curettement should precede this rubbing in of the acid. Another very efficacious remedy is *phenolum sulphuricum*, introduced by Ruault and recommended by Heryng and Przedborski. We apply it with a brush in 20 to 40 per cent. solution. Sendziak considers this drug especially suitable for the treatment of ulceration—not too extensive—of the vocal cords, and the posterior wall of the larynx, and is less useful in treating lesions, such as extensive infiltration of the epiglottis. Another remedy, which seems to act well, is parachloro-phenol applied in

5 to 10 per cent. solution in glycerine. It was introduced by Simanowski in 1894, and is endorsed by Spengler and Logucki. Yet Sendziak cannot report that his own experiences with this drug have been too favourable, the vomiting it provokes being an obstacle to its use. Menthol (10 to 20 per cent.), phenol, creasote, balsamum peruvianum with collodium (1 to 5 per cent.), resorcin (10 to 20 per cent.), peroxide of hydrogen (50 per cent.), perchloride of mercury (1-1,000), liquosulphite, chromic acid, pagol tanin, iodoform (8 per cent.), chloride of zinc injections, electric light (photo-therapy), thiocol (0·1 to 0·15 per cent.), together with other remedies, are merely mentioned by Sendziak, who thinks they might all be dispensed with. Finally Sendziak lays stress on general treatment, especially the great advantages that may often be derived from climates which afford sufficient humidity and are absolutely free from dust as well as rapid changes of temperature. More recently a discussion on the local treatment of laryngeal tuberculosis was opened by Middlemass Hunt [15] at the annual meeting of the British Medical Association. In his opinion neither lactic acid nor any other application would remove an infiltration covered by unbroken mucous membrane. With well-defined, or at least limited, infiltration without ulceration, a mass of granulations or a distinct tumour formation, the first step must be thorough removal of the diseased tissue by the curette, followed by application of lactic acid. The cases suitable for curative treatment were those of limited disease and of hypertrophic form indicating local resistance; cases in which the lung disease was slight and stationary or only very slowly progressive, the general health and appetite good, the patient free from fever, and of a courageous and hopeful temperament. At the same meeting the value of submucous injections into localised tuberculous infiltrations was emphasised by Watson Williams,[16] who mentioned cases illustrating the great value of this method in a limited class of cases.

**Laryngeal Neoplasms.** — *Recurrent papillomata* have been treated by Bronner [17] with local applications of formalin with apparent success. The patient was a male, age 45 years. Both vocal cords were covered with typical papillomata. These were removed with forceps every two or three months, and various local remedies—*e.g.* lactic acid, nitrate of silver, etc.—had been tried, but did not succeed, though subsequent local applications with formalin had seemed to prevent recurrence. An extremely interesting case was shown at the Laryngological Society's meeting in London on May 3rd by Mark Hovell.[18] In 1886 the patient, a male, aged 46, presented a large growth of whitish colour which almost filled the larynx. It was removed through the mouth by forceps, and was found to have been attached to the inner border and under surface of the left vocal cord along its whole length. The history of the patient showed that this growth had been large enough to cause dyspnœa in the summer of 1884. In May 1887 he was again seen by Hovell, when the recurrence of the growth was almost exactly similar to his first observed condition. Again the growth was removed with cutting forceps through the mouth, and six months later there was no recurrence, and that fortunate result was maintained when the patient was shown this year. Mr. F. Eve examined microscopically fragments of the first and second growth; the first he pronounced to be epithelioma with a markedly papillary surface, the second differed from that of the previous year in that it contained no cell nests. But he states that from a histological point of view he had no hesitation in saying that the growth was an epithelioma. It is needless to say the clinical history almost excludes the possibility of the growth having been malignant at all.

[11] Merck's Archiv., May, 1901. [12] Cited Jour. of American Med. Assoc., XXXV., No. 6. [15] Ibid. [14] Jour. of Laryng., May, 1901. [15] Lancet, Aug. 17, 1901. [16] Ibid. [17] Ibid. [18] Jour. of Laryng., June, 1901.

